# Case Report of Subanesthetic Intravenous Ketamine Infusion for the Treatment of Neuropathic Pain and Depression with Suicidal Features in a Pediatric Patient

**DOI:** 10.1155/2018/9375910

**Published:** 2018-07-26

**Authors:** Garret Weber, JuHan Yao, Shemeica Binns, Shinae Namkoong

**Affiliations:** Department of Anesthesiology, New York Medical College, Valhalla, NY, USA

## Abstract

Chronic neuropathic pain and depression are often comorbid. Ketamine has been used to treat refractory pain. There is emerging evidence for use in depression. We present a case of a pediatric patient who was successfully treated with subanesthetic intravenous ketamine infusion for chronic neuropathic pain and suicidality.

## 1. Introduction

Neuropathic pain is often difficult to treat despite multimodal analgesia. Similarly, refractory depression has limited treatment options available. Intravenous ketamine infusion has been increasingly used for the treatment of neuropathic pain. Several recent trials have also shown that intravenous ketamine infusion has a rapid antidepressant effect [1, 2]. There are especially limited research and evidence on the efficacy of the dual treatment of major depression and neuropathic pain in the pediatric population. We present a case of an adolescent with severe depression, suicidality, and neuropathic leg pain who failed multiple antidepressant and analgesic modalities who was successfully treated with subanesthetic intravenous ketamine infusion. To our knowledge this is the first reported case with dual use of intravenous ketamine infusion to treat neuropathic pain and suicidal depression in the pediatric patient.

The patient's healthcare proxy (mother) provided written consent for publication as the patient is a minor.

## 2. Case Description

A 14-year-old female presented to an outpatient psychiatrist with severe depression and suicidal ideation. She was admitted to the pediatric psychiatric unit for evaluation and treatment.

Her medical history was significant for anxiety, depression with multiple suicide attempts, postconcussive syndrome, chronic migraines, and previous diagnosis of complex regional pain syndrome of the lower extremities. While being admitted for depression and suicidality, she also complained of exacerbation of her bilateral leg pain, which significantly limited her mobility and worsened her mood.

She was admitted two months prior for bilateral generalized neuropathic leg pain which limited her mobility. A lumbar magnetic resonance image (MRI) was unremarkable. She tried multiple classes of pain medications including acetaminophen, nonsteroidal anti-inflammatory agents, tricyclic antidepressants, gabapentinoids, antiepileptics, and opioids. Additional interventions included acupuncture, physical therapy, occupational therapy, guided imagery therapy, and epidural steroid injection.

Upon readmission for suicidality, the pain management team was consulted. On her psychiatric evaluation, patient had a depressed, flat affect endorsing suicidality. The patient reported despair regarding her chronic pain as well as flashbacks to a previous concussion after a fall several years prior with subsequent development of her neuropathic type pain. She had also reported several instances of self-injurious behaviors including cutting and two suicide attempts with a shoelace and pillowcase. She was placed on continuous observation. The patient's chronic outpatient psychiatric medications include fluoxetine and aripiprazole. While she was an inpatient, the patient was also trialed on bupropion but was discontinued due to increased agitation and irritability. She also reported severe burning bilateral leg pain. In addition, she described her pain in terms of “shooting”, reporting painful paresthesias, dysesthesias, and hyperalgesia as well as a “numbness” characterization to her bilateral leg pain, which followed a neuropathic pattern.

Her lumbar spine was mildly tender to palpation, but her neurological exam was otherwise intact. While her characterization of pain was neuropathic in nature, she did not meet the criteria for complex regional pain syndrome. There were no allodynic features, vasomotor, pseudomotor, or tropic changes on exam. Despite her prescribed regimen of pregabalin, ibuprofen, acetaminophen, lidocaine 5% patch, tizanidine, and subsequently oxycodone as needed, her pain continued. She had also been tried previously on morphine with also minimal efficacy. The patient also underwent a ten-day trial of duloxetine with minimal improvement and reported worsening severe depressive symptoms, suicidal ideations, and neuropathic pain. It was then recommended to start an intravenous ketamine infusion which was theorized to have a more rapid clinical onset and effect.

Patient was transferred to the pediatric intensive care unit for intravenous ketamine infusion and monitoring. Prior to starting intravenous ketamine infusion, her leg pain was rated 7/10 on the numerical rating scale (NRS, 0-10 with 10 being the worst pain) of burning quality. There was no weakness or changes in sensation, though there was limited mobility secondary to pain. Intravenous ketamine infusion was started at 7 micrograms/kilogram/minute (mcg/kg/min). The ketamine infusion was used 24 hours per day for the entire duration of the treatment with ketamine infusion. The total dose was titrated based on the specific mcg/kg/min. The patient's weight (72 kg) was used for the dosing calculation.

The patient remained hemodynamically stable with no dysphoria or hallucinations. On day one of intravenous ketamine infusion, the patient had significant improvement of her depressive symptoms, as noted by psychiatry and pain management teams, and the maximum NRS throughout the day was 6/10. On day two, she reported less pain in her legs with a maximum NRS of 5/10, and significant improvement in her mood with no further suicidal ideations. On day three, intravenous ketamine infusion was increased to 8 micrograms/kilogram/minute; however physical exam revealed nystagmus and visual changes and was decreased to 7 micrograms/kilogram/minute. Patient's pain maximum NRS score remained at 5/10. On day four, maximum NRS score reduced to 4/10 and self-reported 70% pain relief since initiation of intravenous ketamine infusion. She also had functional improvement in her legs and was able to ambulate freely. On day five, dosage was titrated down to 4 micrograms/kilogram/minute with maximum NRS score of 0/10 and sustained improvement in mood. Intravenous ketamine infusion was further reduced to 2 micrograms/kilogram/minute and discontinued that same day. She was able to tolerate physical therapy and maintain analgesia. Psychiatric reassessment determined her to be no longer at suicide risk, and she was discharged with no immediate sequelae and was placed on chronic aripiprazole, topiramate, and lithium by her outpatient psychiatrist.

The patient had five months of symptom relief after her first intravenous ketamine infusion. She was readmitted five months later with repeated suicide attempts, depression, and worsening bilateral upper and lower extremity neuropathic pain, though with decreased baseline NRS. Due to her dramatic improvement and sustained relief with intravenous ketamine infusion during her last admission, as well as the lack of other successful treatment options, she was again admitted to the pediatric intensive care unit for five days of intravenous ketamine infusion with resolution of her neuropathic pain and suicidal/depressive symptoms allowing for discharge home without any immediate sequelae.

## 3. Discussion

To our knowledge this is the first reported successful pediatric use of intravenous ketamine infusion for the dual treatment of depression with suicidality and chronic neuropathic pain. There is one recent report on the use of ketamine for chronic pain and depression in an adult, but none in the pediatric population [1].

In this case the diagnosis of neuropathic pain was made based on clinical descriptors and history. As noted in a recent review, by Gilron et al., there is no absolute pathognomonic sign or symptom of neuropathic pain [3]. Consequently the patient's diagnosis was made based on her descriptors and symptoms in setting of persistent pain that seemed to reflect a neuropathic pain pattern rather than a nociceptive, nonneuropathic painful condition. The authors of this case acknowledge that there are screening tools used for diagnosis of neuropathic pain and quantitative objective sensory testing. These were not used in our patient, especially in light of her ongoing psychiatric, severe, comorbid symptoms.

Neuropathic pain is nerve pain secondary to peripheral or central nervous injury or dysfunction. It often presents as spontaneous burning or lancinating pain. Other symptoms include allodynia, hyperesthesia, hyperalgesia, and the symptoms are typically persistent even after the primary insult has resolved. The current management for the treatment of neuropathic pain is limited due to minimal evidence of efficacy. Traditional first-line agents include gabapentinoids, tricyclic antidepressants, or serotonin-norepinephrine reuptake inhibitors (SNRIs) [4].

Opioids have had mixed efficacy for neuropathic pain. The evidence supporting use for chronic noncancer pain in children and adolescents is low and limited at best [5]. Furthermore, opioids are considered second/third-line therapy because of their questionable long-term efficacy as well as the risks of opioid related adverse effects which commonly include sedation, gastrointestinal effects including constipation and nausea/vomiting. Additionally respiratory depression can occur. Also, opioid misuse and addiction potential is a serious consideration [3]. Given our patient's comorbid advanced psychiatric state, chronic opioid therapy and further titration were not deemed to be a safe and viable option.

Combination therapy is often required due to nonefficacy of monotherapy. Additional nonpharmacological treatment includes cognitive behavioral therapy, physical therapy, biofeedback, and transcutaneous electrical nerve stimulation (TENS) [6]. Despite a multimodal treatment approach, neuropathic pain can be refractory, and multiple novel targets for neuropathic pain are being studied [7].

The mechanism of neuropathic pain differs for peripheral and central pain. Peripheral sensitization occurs due to an injury to axons of a peripheral nerve. This insult leads to spontaneous activation, abnormal excitability, and increased sensitivity to chemical, mechanical, and thermal stimuli [8]. When this occurs in the spinothalamic tract, central sensitization occurs with increased spontaneous activity and increased responses to all afferent impulses. This increase in synaptic efficiency and reduced inhibition lead to a greater response to nociceptive stimuli [8, 9]. Central sensitization has been proposed as an explanation for persistent neuropathic pain [10]. There is also the “windup” phenomenon in which repetitive nociceptive stimuli are potentiated in the spinal cord causing increased cortical perception of pain [11].

Ketamine is an N-methyl-D-aspartate (NDMA) receptor antagonist. The NMDA receptors are mechanistically involved with central sensitization [8–10]. Ketamine's inhibition of the NMDA receptors helps decrease peripheral and central sensitization, promoting analgesia. In addition, NMDA receptors are involved in the development of opioid tolerance. Ketamine's antagonistic action on NMDA receptors may prevent opioid tolerance in chronic pain patients. Additional mechanisms of ketamine as an analgesic include its effect on substance P. Ketamine has been shown to directly inhibit substance P receptors, which is also increased in painful hyperalgesic states [12].

The mechanistic role of ketamine in the treatment of depression, however, is less well defined. First-line treatment for depression is a combination of psychotherapy with an antidepressant, generally a selective serotonin reuptake inhibitor (SSRI) based on efficacy and side effect profile [13]. Additional classes of medications such as serotonin-norepinephrine reuptake inhibitors, atypical antidepressants, tricyclic antidepressants, or monoamine oxidase inhibitors are often used to treat depression refractory to first-line agents. Electrical convulsive therapy (ECT) is another treatment modality for severe depression refractory to pharmacologic agents. While ECT provides a rapid response and thus has utility in the acute setting, it requires general anesthesia and has complications including somatic injury and dental trauma, as well as confusion and amnesia [14].

As an alternative, ketamine has emerged for treatment of major depressive disorder with or without suicidality. Several trials demonstrated a large reduction in the severity of depression within 24 hours of intravenous ketamine infusion with lasting antidepressant efficacy [2]. A systematic literature review also demonstrated reduction in suicidal ideation for treatment resistant depression with the use of ketamine [15]. An additional benefit is the rapid antidepressant effect compared with most first-line antidepressant medications.

The antidepressant mechanism of ketamine is unclear at this point but may involve ketamine's NMDA antagonism. In fact, citalopram and fluoxetine, both SSRIs, have been shown to have functional effects of NMDA receptor blockade [16]. Ketamine, by inhibiting NMDA receptors, leads to increased production of brain-derived neurotrophic factor (BDNF) and mammalian target of rapamycin complex 1 (mTORC1), both of which are associated with synaptogenesis and have been shown to be decreased in patients with major depression [16].

One limitation of using ketamine is the higher level monitoring. Due to state regulations, at our institution, patients receiving an intravenous ketamine infusion require an intensive care unit or step down unit for the first 24 hours; it is therefore a resource burden [17]. Furthermore, the long-term cognitive effects and optimal subanesthetic dosing and duration for the dual treatment of neuropathic pain and depression need to be elucidated. Additionally, the described subanesthetic intravenous ketamine infusion is an off-label use in pediatric neuropathic pain and depression.

In summary, we have shown successfully in our trial that subanesthetic intravenous ketamine infusion has the potential for emerging use in the pediatric population for neuropathic pain and depression given its rapid-onset antidepressant and analgesic effects as well as favorable safety profile at subanesthetic dosing.
